# Survival after standard or oncoplastic breast-conserving surgery *versus* mastectomy for breast cancer

**DOI:** 10.1093/bjsopen/zraf002

**Published:** 2025-03-19

**Authors:** Mhairi Mactier, James Mansell, Laura Arthur, Julie Doughty, Laszlo Romics

**Affiliations:** Wolfson Wohl Cancer Research Centre, College of Medicine and Veterinary Science, University of Glasgow, Glasgow, UK; General Surgery Department, Golden Jubilee National Hospital, Clydebank, UK; General Surgery Department, Gartnavel General Hospital, Glasgow, UK; General Surgery Department, Royal Alexandria Hospital, Paisley, UK; General Surgery Department, Gartnavel General Hospital, Glasgow, UK; General Surgery Department, Gartnavel General Hospital, Glasgow, UK

## Abstract

**Background:**

Recent evidence suggests a survival advantage after breast-conserving surgery compared with mastectomy. Previous studies have compared survival outcomes after standard breast-conserving surgery, but no studies have compared survival outcomes after oncoplastic breast-conserving surgery. The aim of this study was to compare survival outcomes after breast-conserving surgery + radiotherapy (and an oncoplastic breast-conserving surgery + radiotherapy subgroup) with those after mastectomy ± radiotherapy.

**Methods:**

Patients diagnosed with primary invasive breast cancer between 1 January 2010 and 31 December 2019 were identified from a prospectively maintained National Cancer Registry. Overall survival and breast cancer-specific survival outcomes were analysed using Kaplan–Meier analysis and Cox regression analysis adjusting for patient demographics, tumour characteristics, and treatment adjuncts.

**Results:**

A total of 14 182 patients were eligible (8537 patients underwent standard breast-conserving surgery + radiotherapy, 360 patients underwent oncoplastic breast-conserving surgery + radiotherapy, 2953 patients underwent mastectomy + radiotherapy, and 2332 patients underwent mastectomy − radiotherapy). The median follow-up was 7.27 (range 0.2–13.6) years. Superior 10-year survival was observed after breast-conserving surgery + radiotherapy (overall survival: 81.2%; breast cancer-specific survival: 93.3%) compared with mastectomy + radiotherapy (overall survival: 63.4%; breast cancer-specific survival: 75.9%) and mastectomy − radiotherapy (overall survival: 63.1%; breast cancer-specific survival: 87.5%). Ten-year overall survival and breast cancer-specific survival after oncoplastic breast-conserving surgery + radiotherapy were 86.1% and 90.2% respectively. After adjusted analysis, breast-conserving surgery + radiotherapy was associated with superior survival outcomes compared with mastectomy + radiotherapy (overall survival: HR 1.34 (95% c.i. 1.20 to 1.51); breast cancer-specific survival: HR 1.62 (95% c.i. 1.38 to 1.90)) and mastectomy − radiotherapy (overall survival: HR 1.57 (95% c.i. 1.41 to 1.75); breast cancer-specific survival: HR 1.70 (95% c.i. 1.41 to 2.05)). Similar survival outcomes were observed amongst patients treated with oncoplastic breast-conserving surgery + radiotherapy compared with mastectomy + radiotherapy (overall survival: HR 1.72 (95% c.i. 1.62 to 2.55); breast cancer-specific survival: HR 1.74 (95% c.i. 1.06 to 2.86)) and mastectomy − radiotherapy (overall survival: HR 2.21 (95% c.i. 1.49 to 3.27); breast cancer-specific survival: HR 1.89 (95% c.i. 1.13 to 3.14)).

**Conclusion:**

Breast-conserving surgery + radiotherapy and oncoplastic breast-conserving surgery + radiotherapy are associated with superior overall survival and breast cancer-specific survival compared with mastectomy ± radiotherapy. The findings should inform discussion of surgical treatment options for patients with breast cancer.

## Teaser

Further evidence to support survival benefit associated with conservation surgery and radiotherapy compared with mastectomy. This article demonstrates similar survival benefit amongst a subgroup of patients undergoing oncoplastic breast-conserving surgery and the findings should guide conversations with breast cancer patients regarding treatment options.

## Introduction

Breast cancer affects one in seven UK women^1^, with surgical intervention the mainstay of treatment. Historically, women underwent radical mastectomy (Mx) regardless of tumour extent or size. Pioneering work in the 1970s shifted the paradigm towards a more conservative approach^2,3^, with large randomized clinical trials (RCTs) demonstrating comparable long-term survival outcomes after breast-conserving surgery (BCS) + radiotherapy (RTx) compared with Mx^4–6^. More recent reviews suggest BCS + RTx is associated with superior overall survival (OS) and breast cancer-specific survival (BCSS)^7–9^, with survival benefit maintained after adjustment for tumour biology, cancer staging, socio-economic status, and co-morbidities^10–13^.

Most previously published studies only include T1–T2 cancers and provide no information regarding oncoplastic breast-conserving surgery (OBCS), which has become the standard of care, blending oncological and plastic surgical techniques to improve long-term aesthetic outcomes and quality of life^14^. OBCS shifts BCS towards larger tumours, enabling a subgroup of patients who would have previously been selected for Mx to avoid removal of the entire breast. Breast cancers detected through screening are associated with a better prognosis^12,15^, but few studies assessing survival outcomes have taken account of the mode of patient referral (screening or symptomatic). Furthermore, various cancer registries report differing effects of breast conservation upon prognoses. In the absence of an existing UK-based population study, it is important to investigate whether BCS has a similar survival advantage in Scotland.

The primary aim of this study was to evaluate and compare survival outcomes after BCS + RTx and Mx ± RTx in the West of Scotland population, taking account of the mode of patient referral, as well as other demographic and prognostic factors. The secondary aim of this study was to provide additional understanding of the survival outcomes after OBCS + RTx.

## Methods

### Study design

#### Population

The population consisted of all patients diagnosed with breast cancer in the West of Scotland over a 10-year interval (between 1 January 2010 and 31 December 2019).

#### Data collection

Prospective data for all patients diagnosed with breast cancer in the West of Scotland are collected by clinical audit facilitators from each NHS Scotland health board. Data are entered locally into the electronic Cancer Audit Support Environment (eCASE) National Cancer Registry, which is managed by the West of Scotland Cancer Network (WoSCAN). WoSCAN is a collaborative of four NHS health boards (NHS Ayrshire & Arran, NHS Forth Valley, NHS Greater Glasgow & Clyde, and NHS Lanarkshire).

In addition to patient demographics, data collected include date of diagnosis, whether the cancer was detected through screening or symptomatic (mode of referral), laterality, clinical staging at presentation, histopathology and tumour biology, date and type of surgery, and any treatment adjuncts received^16^.

Type of surgery was recorded as standard BCS (sBCS), OBCS, or Mx with or without immediate breast reconstruction. OBCS, defined by the operating surgeon, was collected as a separate surgical code from 2014. OBCS procedures were exclusively level two procedures, according to Clough *et al*.^17^, with tumour size requiring resection of more than 20% of the total breast volume. Procedures included therapeutic mammoplasty or mastopexy and chest wall perforator flap partial breast reconstruction. An additional 65 OBCS procedures carried out between 2010 and 2014 were taken from a separate prospectively collected regional database and added to the main database manually. The patients who underwent OBCS were included in the breast conservation group (BCS + RTx) to address the primary outcome and, to address the secondary outcome, involving the analysis of survival outcomes after OBCS + RTx, patients who underwent sBCS were excluded. Mx with immediate breast reconstruction was collected as a separate surgical code from 2012. These patients were included in the Mx group (Mx ± RTx). Data for RTx site(s) and dosage were not sufficiently available and therefore adjuvant RTx was treated as a binary variable (yes/no).

In the WoSCAN database, clinical and pathological staging are recorded according to the AJCC’s TNM classification of malignant tumours^18,19^. To assess status variables unaffected by treatment, pretreatment clinical variables (cT and cN) were used in the analysis of patients who received neoadjuvant treatment, whereas histopathological variables were used in the analysis of patients who underwent primary surgery (pT and pN). Similarly, data on tumour biology were based on pretreatment core biopsy and histopathological tumour specimen respectively, unless the test was repeated on the surgical specimen. Oestrogen receptor (ER) status was reported using the Allred system^20^; scores less than or equal to two were considered negative. Human epidermal growth factor receptor-2 (HER2) amplification was confirmed by an immunohistochemistry (IHC) score of greater than or equal to three or by *in situ* hybridization, which was performed when the HER2 IHC score was greater than or equal to two. Hormone receptor subgroups were classified as ER+HER2+, ER+HER2−, ER−HER2+, and ER−HER2−. Progesterone receptor (PR) status was not collected before 2016 and so was not included as a variable in the analysis.

Socio-economic deprivation was calculated from each patient’s residential postcode using the Scottish Index of Multiple Deprivation (SIMD) 2020 (version 2)^21^. This tool divides Scotland into 6976 data zones, which are then categorized into quintiles, from most deprived (SIMD 1) to least deprived (SIMD 5). Date and cause of death were obtained from the Acute, Cancer, Deaths, and Mental Health (ACaDMe) data mart, part of the NHS National Scotland (NSS) Corporate Data Warehouse. Records were linked using the personal identification (community health index (CHI)) numbers assigned to all Scottish residents.

#### Inclusion criteria

Inclusion criteria were: primary invasive breast cancer diagnosed between 2010 and 2019; and patients treated with BCS + RT, Mx + RTx, or Mx − RTx.

#### Exclusion criteria

Exclusion criteria were: *in situ* disease or unknown tumour subtype; stage IV disease (‘any surgical intervention received in this group was considered palliative’) or unknown clinical stage; missing data for type of surgery or adjuvant RTx; patients treated with BCS − RTx; and unknown date of death or date recorded precedes study interval.

### Ethical considerations

The Regional Information Government Framework at WoSCAN safeguards sharing of data for clinical audit purposes^22^. All analysis was performed on pseudonymized data. Principles of Good Clinical Practice and the Declaration of Helsinki were adhered to.

### Statistical methods

Start of follow-up was from the date of diagnosis until death or the end of the study interval on 1 August 2023. Death attributable to any cause (all-cause mortality) and death attributed to breast cancer (breast cancer-specific mortality) were assessed. Differences in patient and tumour characteristics between treatment groups were analysed using the chi-squared test. Unadjusted survival proportions were estimated using Kaplan–Meier analysis and compared using the log rank test. OS and BCSS were modelled and adjusted using Cox regression analysis. Associations between treatment groups and survival rates are reported as HR (95% c.i.). All tests were two-sided and *P* < 0.050 was considered significant. Models were stepwise adjusted for variables, including age at diagnosis, year of diagnosis, mode of referral, socio-economic deprivation, tumour characteristics, clinical staging, and treatment adjuncts. All statistical analysis were carried out using SPSS^®^ (IBM, Armonk, NY, USA; version 29.1; https://www.ibm.com/products/spss-statistics).

## Results

**Fig. 1.**
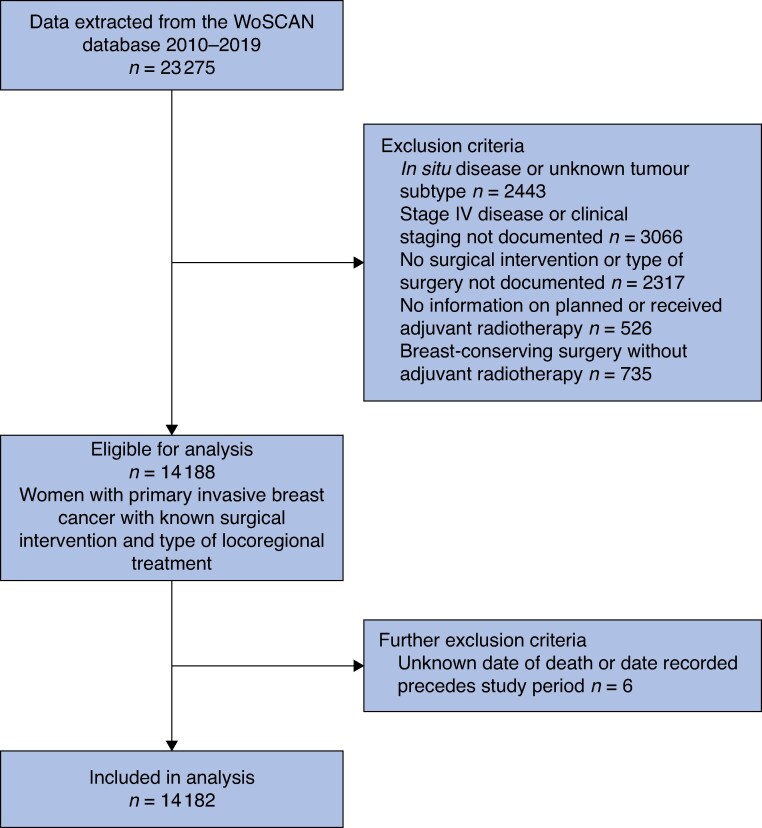
CONSORT diagram for all breast cancer diagnoses in the West of Scotland between 2010 and 2019 WoSCAN, West of Scotland Cancer Network.

A total of 23 275 patients were diagnosed and treated for breast cancer across the West of Scotland between 2010 and 2019; 14 182 patients met the inclusion criteria for the final analysis (*Fig. 1*).

Some 8897 (62.7%) patients underwent BCS + RTx, 2953 (20.9%) patients underwent Mx + RTx, and 2332 (16.4%) patients underwent Mx − RTx. Amongst the breast conservation group, 8537 (60.2%) patients underwent sBCS + RTx and 360 (2.5%) patients underwent OBCS + RTx. The median follow-up was 7.27 (range 0.2–13.6) years. See *Table 1* for patient and tumour characteristics by treatment modality.

**Table 1:** Patient/tumour characteristics by treatment modality (*n* = 14 182)

Patient/tumour characteristic	Treatment modality					*P*
	sBCS + RTx, 8537 (60.2)	OBCS + RTx, 360 (2.5)	Mx + RTx, 2953 (20.9)	Mx − RTx, 2332 (16.4)	Total, *n* = 14 182	
**Age at diagnosis (years)**						<0.001
<50	1412 (16.5)	118 (32.8)	882 (29.9)	336 (14.4)	2748 (19.3)	
50–69	5625 (65.9)	220 (61.1)	1374 (46.5)	1045 (44.8)	8264 (58.3)	
≥70	1500 (17.6)	22 (6.1)	697 (23.6)	951 (40.8)	3170 (22.4)	
Median (range)	61 (23–92)	53 (23–80)	57 (25–94)	67 (22–96)	61 (22–96)	
**Referral**						<0.001
Symptomatic	4135 (48.4)	237 (65.8)	2330 (78.9)	1493 (64.0)	8195 (57.8)	
Screening	3875 (45.4)	100 (27.8)	394 (13.3)	543 (23.3)	4912 (34.6)	
Other	527 (6.2)	23 (6.4)	229 (7.8)	296 (12.7)	1075 (7.6)	
**Year of diagnosis**						<0.001
2010–2011	1022 (12.0)	28 (7.8)	423 (14.3)	364 (15.6)	1837 (13.0)	
2012–2013	1887 (22.1)	39 (10.8)	714 (24.2)	566 (24.3)	3206 (22.6)	
2014–2015	1908 (22.3)	98 (27.2)	655 (22.2)	542 (23.2)	3203 (22.6)	
2016–2017	1815 (21.3)	103 (28.6)	595 (20.1)	432 (18.5)	2945 (20.8)	
2018–2019	1905 (22.3)	92 (25.6)	566 (19.2)	428 (18.4)	2991 (21.1)	
**SIMD category**						0.006
Most affluent	1668 (19.6)	90 (25.1)	569 (19.4)	408 (17.5)	2735 (19.4)	
Affluent	1449 (17.1)	64 (17.9)	489 (16.7)	394 (17.0)	2396 (17.0)	
Intermediate	1518 (17.9)	71 (19.8)	505 (17.2)	378 (16.3)	2472 (17.5)	
Deprived	1739 (20.5)	64 (17.9)	608 (20.7)	525 (22.6)	2936 (20.8)	
Most deprived	2116 (24.9)	69 (19.3)	763 (26.0)	615 (26.5)	3563 (25.3)	
**Bilateral breast cancer**						<0.001
No	8421 (98.9)	354 (98.3)	2846 (97.1)	2253 (96.9)	13 874 (98.2)	
Yes	96 (1.1)	6 (1.7)	86 (2.9)	71 (3.1)	259 (1.8)	
T stage*						<0.001
T1	5657 (66.3)	112 (31.3)	462 (16.1)	1040 (44.6)	7271 (51.6)	
T2	2704 (31.7)	209 (58.4)	1578 (54.8)	1201 (51.5)	5692 (40.3)	
≥T3	171 (2.0)	37 (10.3)	837 (29.1)	90 (3.9)	1135 (8.1)	
N stage*						<0.001
N0	6383 (75.0)	255 (70.8)	654 (22.2)	1917 (85.2)	9209 (65.5)	
N1	1867 (21.9)	87 (24.2)	1704 (58.1)	266 (11.8)	3924 (27.9)	
N2	180 (2.1)	13 (3.6)	386 (13.2)	46 (2.0)	625 (4.4)	
N3	83 (1.0)	5 (1.4)	191 (6.5)	22 (1.0)	301 (2.1)	
**Prognostic stage**						<0.001
I	5555 (65.1)	108 (30.0)	408 (13.8)	1009 (43.3)	7080 (49.9)	
II	2791 (32.7)	230 (63.9)	1856 (62.9)	1230 (52.7)	6107 (43.1)	
III	191 (2.2)	22 (6.1)	689 (23.3)	93 (4.0)	995 (7.0)	
**Histological subtype**						<0.001
Ductal	7137 (83.6)	295 (81.9)	2238 (75.8)	1775 (76.1)	11 445 (80.7)	
Lobular	754 (8.8)	48 (13.3)	507 (17.2)	360 (15.4)	1669 (11.8)	
Other	643 (7.5)	17 (4.7)	207 (7.0)	197 (8.4)	1064 (7.5)	
**Tumour grade**						<0.001
1	1287 (15.3)	22 (6.3)	114 (4.1)	218 (9.5)	1641 (11.8)	
2	4035 (47.9)	159 (45.3)	1211 (43.3)	1123 (48.8)	6528 (47.1)	
3	3095 (36.8)	170 (48.4)	1473 (52.6)	959 (41.7)	5697 (41.1)	
**Subtype**						<0.001
ER+HER2−	6157 (72.1)	231 (64.2)	1763 (59.7)	1503 (64.5)	9654 (68.1)	
ER+HER2+	696 (8.2)	52 (14.4)	401 (13.6)	213 (9.1)	1362 (9.6)	
ER−HER2+	274 (3.2)	15 (4.2)	207 (7.0)	113 (4.8)	609 (4.3)	
ER−HER2−	592 (6.9)	18 (5.0)	254 (8.6)	190 (8.1)	1054 (7.4)	
Inconclusive	818 (9.6)	44 (12.2)	328 (11.1)	313 (13.4)	1503 (10.6)	
**Chemotherapy**						<0.001
No	5548 (65.0)	147 (40.8)	863 (29.2)	1767 (75.8)	8325 (58.7)	
Yes	2989 (35.0)	213 (59.2)	2090 (70.8)	565 (24.2)	5857 (41.3)	
**Biological therapy**						<0.001
No	7681 (90.0)	287 (79.7)	2369 (80.2)	2116 (90.7)	12 453 (87.8)	
Yes	856 (10.0)	73 (20.3)	584 (19.8)	216 (9.3)	1729 (12.2)	
**Endocrine therapy**						<0.001
No	1333 (15.6)	61 (16.9)	688 (23.3)	519 (22.3)	2601 (18.3)	
Yes	7204 (84.4)	299 (83.1)	2265 (76.7)	1813 (77.7)	11 581 (81.7)	
**Deaths**						
Overall	1265 (14.8)	37 (10.3)	926 (31.4)	734 (31.5)	2962 (20.9)	<0.001
Breast cancer-specific	450 (5.3)	26 (7.2)	604 (20.5)	246 (10.5)	1326 (9.3)	<0.001

Values are *n* (%) unless otherwise indicated. *Pathological stage used for primary surgery patients and pretreatment clinical stage used for neoadjuvant chemotherapy patients. sBCS, standard breast-conserving surgery; RTx, radiotherapy; OBCS, oncoplastic breast-conserving surgery; MTx, mastectomy; SMID, Scottish Index of Multiple Deprivation; ER, oestrogen receptor; HER2, human epidermal growth factor receptor-2.

A higher proportion of patients in the BCS + RTx group presented through the screening pathway compared with those who underwent Mx + RTx or Mx − RTx (screening: 44.7% *versus* 13.3% (Mx + RTx) and 23.3% (Mx − RTx), *P* < 0.001). Similarly, smaller tumours and early stage I disease were more common in the BCS + RTx group (T1 disease: 64.9% *versus* 16.1% (Mx + RTx) *versus* 44.6% (Mx − RTx), *P* < 0.001; stage I disease: 63.7% *versus* 13.8% (Mx + RTx) *versus* 43.3% (Mx − RTx), *P* < 0.001). In addition, ER+HER2− tumours (associated with a good prognosis) were more common amongst the breast conservation group (*P* < 0.001).

Nodal involvement was more common amongst the patients who underwent Mx + RTx and a higher proportion of this cohort received chemotherapy compared with those who underwent BCS + RTx or Mx − RTx (nodal involvement: 77.8% *versus* 25.2% (BCS + RTx) *versus* 14.8% (Mx − RTx), *P* < 0.001; chemotherapy: 70.8% *versus* 36.0% (BCS + RTx) *versus* 24.2% (Mx − RTx), *P* < 0.001). Lobular cancers were more common amongst the Mx group compared with the BCS + RTx group (*P* < 0.001), which may be reflective of their multifocal nature. In the unadjusted analysis, more deaths were observed in the Mx cohort, consistent with patients presenting with more advanced disease.

In total, 2962 (20.9%) deaths occurred during the follow-up interval, of which 1326 (9.3%) were attributed to breast cancer. Within the BCS + RTx group, 1302 (14.6%) deaths were observed, with 476 (5.3%) attributed to breast cancer; 5-year OS was 92.5% and 5-year BCSS was 96.2%. Ten-year survival rates were 81.2% (OS) and 93.3% (BCSS). Within the Mx + RTx group, there were 926 (31.4%) deaths, with 604 (20.5%) attributed to breast cancer; 5-year survival rates were 79.8% (OS) and 84.5% (BCSS) and 10-year survival rates were 63.4% (OS) and 75.9% (BCSS). Within the Mx − RTx group there were 734 (31.5%) deaths, with 246 (10.5%) attributed to breast cancer; 5-year survival rates were 82.2% (OS) and 92.0% (BCSS) and 10-year survival rates were 63.1% (OS) and 87.5% (BCSS).

**Fig. 2.**
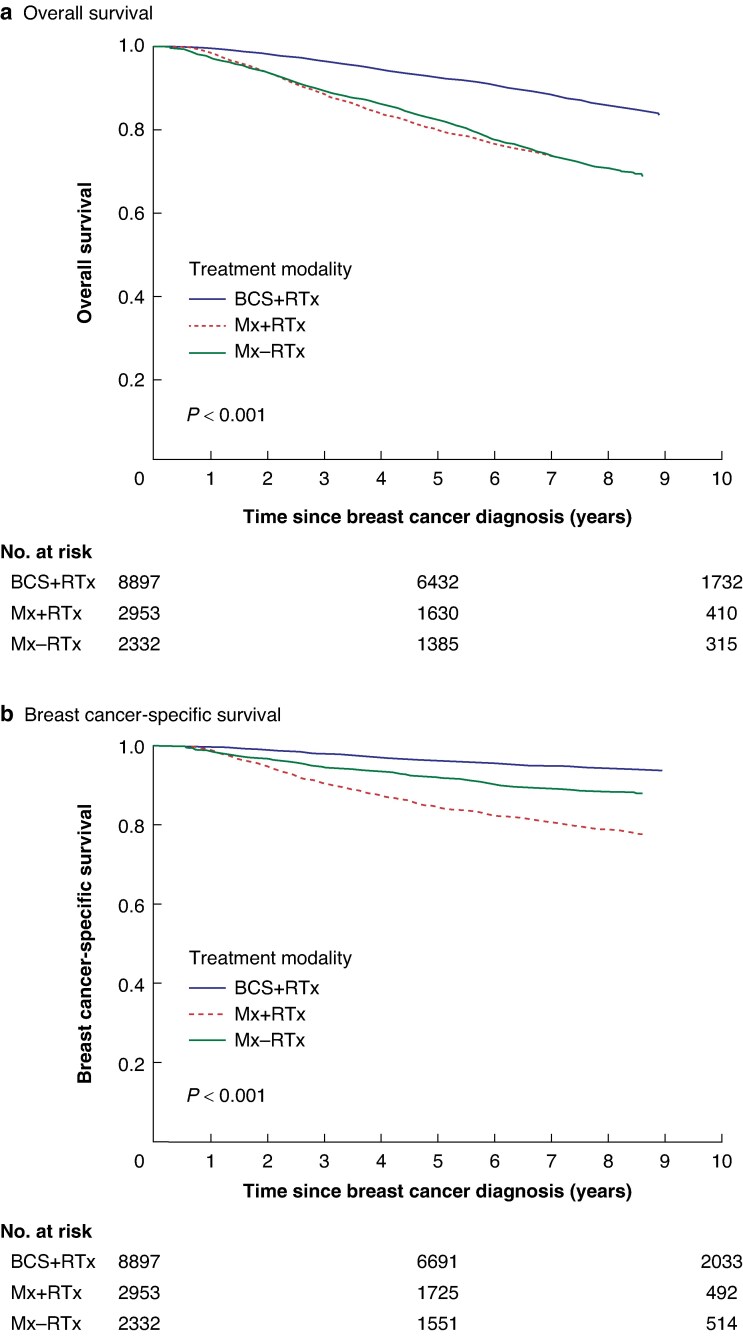
Kaplan–Meier survival curves comparing breast-conserving surgery + radiotherapy and mastectomy ± radiotherapy **a** Overall survival. **b** Breast cancer-specific survival. BCS, breast-conserving surgery; RTx, radiotherapy; MTx, mastectomy.

Superior OS and BCSS were observed after BCS + RTx compared with Mx ± RTx via Kaplan–Meier survival analysis using the log rank test (*Fig. 2*). In unadjusted analysis, Mx ± RTx was associated with the lowest OS (*Fig. 2a*) and Mx + RTx was associated with the lowest BCSS (*Fig. 2b*).

In Cox regression models adjusted for age, year of diagnosis, and mode of referral, BCS + RTx was associated with superior OS and BCSS compared with Mx ± RTx. Superior survival outcomes after BCS + RTx remained significant after further adjustments for socio-economic deprivation, tumour characteristics, staging, and treatment adjuncts (*Table 2*).

**Table 2:** Multivariable analysis using Cox regression to compare breast-conserving surgery + radiotherapy and mastectomy ± radiotherapy—HRs of overall survival and breast cancer-specific survival by treatment modality adjusted stepwise for patient demographics, socio-economic deprivation, tumour characteristics, staging, and treatment adjuncts

	Deaths	HR (95% c.i.), *P*			
		Model A	Model B	Model C	Model D
**Overall survival**					
BCS + RTx	1302 (14.6)	1 (reference)	1 (reference)	1 (reference)	1 (reference)
Mx + RTx	926 (31.4)	1.95 (1.78,2.12), <0.001	1.96 (1.80,2.14), <0.001	1.32 (1.17,1.48), <0.001	1.34 (1.20,1.51), <0.001
Mx − RTx	734 (31.5)	1.59 (1.45,1.75), <0.001	1.60 (1.45,1.75), <0.001	1.67 (1.50,1.86), <0.001	1.57 (1.41,1.75), <0.001
**Breast cancer-specific survival**					
BCS + RTx	476 (5.3)	1 (reference)	1 (reference)	1 (reference)	1 (reference)
Mx + RTx	604 (20.5)	3.18 (2.81,3.60), <0.001	3.22 (2.84,3.64), <0.001	1.61 (1.37,1.89), <0.001	1.62 (1.38,1.90), <0.001
Mx − RTx	246 (10.5)	1.61 (1.38,1.89), <0.001	1.62 (1.38,1.90), <0.001	1.80 (1.49,2.17), <0.001	1.70 (1.41,2.05), <0.001

Values are *n* (%) unless otherwise indicated. Model A: adjusted for age, year of diagnosis, and mode of referral. Model B: adjusted for the same variables as model A + socio-economic deprivation. Model C: adjusted for the same variables as model B + tumour grade, tumour size, receptor profile, and lymph node status (pathological staging for primary surgery patients and clinical staging for neoadjuvant chemotherapy patients). Model D: adjusted for the same variables as model C + treatment adjuncts (chemotherapy, biological therapy, and/or endocrine therapy). BCS, breast-conserving surgery; RTx, radiotherapy; MTx, mastectomy.

In a multivariable analysis, older age at diagnosis, patients living in areas of highest deprivation, increased tumour size, advanced tumour grade (grade 3), unfavourable tumour subtypes (ER+HER2+, ER−HER2+, and ER−HER2−), and axillary lymph node metastasis were associated with inferior OS. Presentation via screening programmes (HR 0.66 (95% c.i. 0.59 to 0.75), *P* < 0.001) and use of treatment adjuncts (chemotherapy: HR 0.68 (95% c.i. 0.60 to 0.76), *P* < 0.001; biological therapy: HR 0.53 (95% c.i. 0.42 to 0.66), *P* < 0.001; endocrine therapy: HR 0.73 (95% c.i. 0.58 to 0.91), *P* = 0.006) were associated with superior OS (*Table 3*). Similar findings were identified for BCSS; patients aged greater than or equal to 70 years at diagnosis, living in areas of highest deprivation, with increased tumour size and advanced tumour grade, with tumour subtypes ER+HER2+ and ER−HER2−, and with axillary lymph node metastasis were associated with inferior BCSS. Presentation via screening programmes (HR 0.63 (95% c.i. 0.52 to 0.77), *P* < 0.001) and use of treatment adjuncts (chemotherapy: HR 0.91 (95% c.i. 0.77 to 1.09), *P* = 0.295; biological therapy: HR 0.41 (95% c.i. 0.30 to 0.57), *P* < 0.001; endocrine therapy: HR 0.52 (95% c.i. 0.38 to 0.71), *P* < 0.001), with the exception of chemotherapy, were associated with superior BCSS (*Table 4*).

**Table 3:** Multivariable analysis table comparing breast-conserving surgery + radiotherapy and mastectomy ± radiotherapy for overall survival adjusted for patient demographics, socio-economic deprivation, tumour characteristics, staging, and treatment adjuncts

Characteristic	Value	HR (95% c.i.)	*P*
**Treatment modality**			
BCS + RTx	8897 (62.7)	1 (reference)	
Mx + RTx	2953 (20.9)	1.34 (1.20,1.51)	<0.001
Mx − RTx	2332 (16.4)	1.57 (1.41,1.75)	< .001
**Age at diagnosis (years)**			
<50	2748 (19.3)	1 (reference)	
50–69	8264 (58.3)	1.45 (1.26,1.66)	<0.001
≥70	3170 (22.4)	3.12 (2.70,3.61)	<0.001
**Referral**			
Symptomatic	8195 (57.8)	1 (reference)	
Screening	4912 (34.6)	0.66 (0.59,0.75)	<0.001
Other	1075 (7.6)	1.17 (1.02,1.35)	0.027
**Year of diagnosis**			
2010–2011	1837 (13.0)	1 (reference)	
2012–2013	3206 (22.6)	0.89 (0.78,1.01)	0.078
2014–2015	3203 (22.6)	0.98 (0.85,1.13)	0.805
2016–2017	2945 (20.8)	0.87 (0.74,1.03)	0.106
2018–2019	2991 (21.1)	0.78 (0.65,0.94)	0.008
**SIMD category**			
Most affluent	2735 (19.4)	1 (reference)	
Affluent	2396 (17.0)	0.96 (0.83,1.11)	0.574
Intermediate	2472 (17.5)	1.09 (0.95,1.26)	0.217
Deprived	2936 (20.8)	1.12 (0.99,1.28)	0.083
Most deprived	3563 (25.3)	1.46 (1.29,1.65)	<0.001
**Tumour grade**			
1	1641 (11.8)	1 (reference)	
2	6528 (47.1)	1.03 (0.87,1.21)	0.741
3	5697 (41.1)	1.48 (1.25,1.76)	<0.001
**Tumour size**			
T1	7271 (51.6)	1 (reference)	
T2	5692 (40.3)	1.29 (1.18,1.42)	<0.001
≥T3	1135 (8.1)	2.06 (1.77,2.40)	<0.001
**Tumour subtype**			
ER+HER2−	9654 (68.1)	1 (reference)	
ER+HER2+	1362 (9.6)	1.30 (1.08,1.57)	0.006
ER−HER2+	609 (4.3)	1.41 (1.05,1.89)	0.021
ER−HER2−	1054 (7.4)	1.42 (1.11,1.82)	0.005
**Lymph node status**			
N0	9209 (65.5)	1 (reference)	
N1–3	4850 (34.5)	1.63 (1.48,1.80)	<0.001
**Chemotherapy**			
No	8325 (58.7)	1 (reference)	
Yes	5857 (41.3)	0.68 (0.60,0.76)	<0.001
**Biological therapy**			
No	12 453 (87.8)	1 (reference)	
Yes	1729 (12.2)	0.53 (0.42,0.66)	<0.001
**Endocrine therapy**			
No	2601 (18.3)	1 (reference)	
Yes	11 581 (81.7)	0.73 (0.58,0.91)	0.006

Values are *n* (%) unless otherwise indicated. BCS, breast-conserving surgery; RTx, radiotherapy; Mx, mastectomy; SMID, Scottish Index of Multiple Deprivation; ER, oestrogen receptor; HER2, human epidermal growth factor receptor-2.

**Table 4:** Multivariable analysis table comparing breast-conserving surgery + radiotherapy and mastectomy ± radiotherapy for breast cancer-specific survival adjusted for patient demographics, socio-economic deprivation, tumour characteristics, staging, and treatment adjuncts

Characteristic	Value	HR (95% c.i.)	*P*
**Treatment modality**			
BCS + RTx	8897 (62.7)	1 (reference)	
Mx + RTx	2953 (20.9)	1.62 (1.38,1.90)	<0.001
Mx − RTx	2332 (16.4)	1.70 (1.41,2.05)	<0.001
**Age at diagnosis (years)**			
<50	2748 (19.3)	1 (reference)	
50–69	8264 (58.3)	0.99 (0.84,1.17)	0.947
≥70	3170 (22.4)	1.35 (1.11,1.65)	0.003
**Referral**			
Symptomatic	8195 (57.8)	1 (reference)	
Screening	4912 (34.6)	0.63 (0.52,0.77)	<0.001
Other	1075 (7.6)	1.38 (1.12,1.70)	0.003
**Year of surgery**			
2010–2011	1837 (13.0)	1 (reference)	
2012–2013	3206 (22.6)	0.82 (0.67,1.01)	0.064
2014–2015	3203 (22.6)	0.87 (0.71,1.08)	0.210
2016–2017	2945 (20.8)	0.80 (0.62,1.02)	0.071
2018–2019	2991 (21.1)	0.70 (0.54,0.91)	0.008
**SIMD category**			
Most affluent	2735 (19.4)	1 (reference)	
Affluent	2396 (17.0)	0.90 (0.72,1.12)	0.335
Intermediate	2472 (17.5)	0.97 (0.78,1.20)	0.776
Deprived	2936 (20.8)	1.10 (0.90,1.34)	0.360
Most deprived	3563 (25.3)	1.34 (1.11,1.60)	0.002
**Tumour grade**			
1	1641 (11.8)	1 (reference)	
2	6528 (47.1)	2.65 (1.62,4.34)	<0.001
3	5697 (41.1)	5.38 (3.29,8.81)	<0.001
**Tumour size**			
T1	7271 (51.6)	1 (reference)	
T2	5692 (40.3)	1.96 (1.67,2.32)	<0.001
≥T3	1135 (8.1)	3.37 (2.72,4.17)	<0.001
**Tumour subtype**			
ER+HER2−	9654 (68.1)	1 (reference)	
ER+HER2+	1362 (9.6)	1.51 (1.12,2.02)	0.007
ER−HER2+	609 (4.3)	1.06 (0.69,1.62)	0.787
ER−HER2−	1054 (7.4)	1.41 (1.01,1.97)	0.042
**Lymph node status**			
N0	9209 (65.5)	1 (reference)	
N1–3	4850 (34.5)	2.37 (2.04,2.76)	<0.001
**Chemotherapy**			
No	8325 (58.7)	1 (reference)	
Yes	5857 (41.3)	0.91 (0.77,1.09)	0.295
**Biological therapy**			
No	12 453 (87.8)	1 (reference)	
Yes	1729 (12.2)	0.41 (0.30,0.57)	<0.001
**Endocrine therapy**			
No	2601 (18.3)	1 (reference)	
Yes	11 581 (81.7)	0.52 (0.38,0.71)	<0.001

Values are *n* (%) unless otherwise indicated. BCS, breast-conserving surgery; RTx, radiotherapy; Mx, mastectomy; SMID, Scottish Index of Multiple Deprivation; ER, oestrogen receptor; HER2, Human epidermal growth factor receptor-2.

**Fig. 3:**
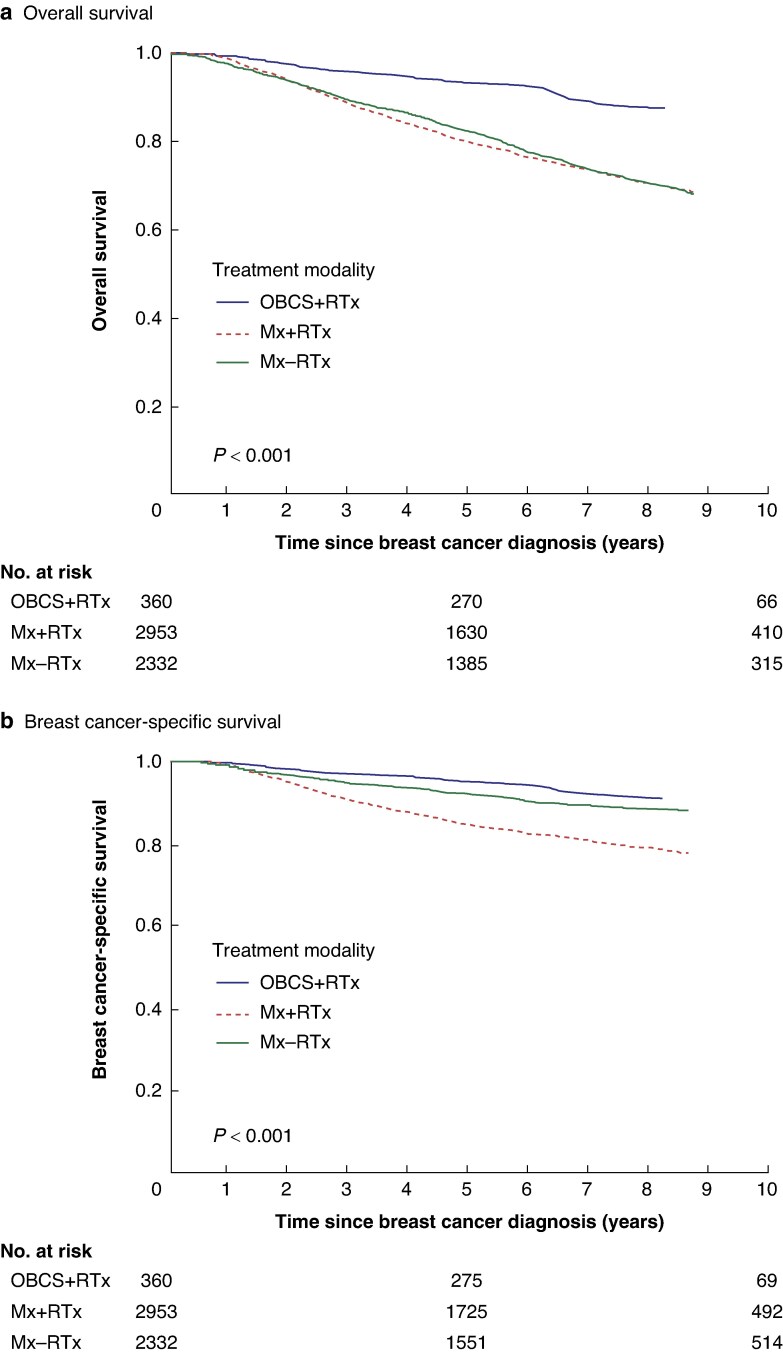
Kaplan–Meier survival curves comparing oncoplastic breast-conserving surgery + radiotherapy and mastectomy ± radiotherapy **a** Overall survival. **b** Breast cancer-specific survival. OBCS, oncoplastic breast-conserving surgery; RTx, radiotherapy; MTx, mastectomy.

To better understand the survival outcomes associated with OBCS + RTx, subgroup analysis was carried out comparing survival outcomes after OBCS + RTx and M ± RTx (5645 patients), excluding patients who underwent sBCS + RTx. Within the OBCS + RTx group (360 patients), 37 (10.3%) deaths were observed, with 26 (7.2%) attributed to breast cancer; 10-year survival rates were 86.1% (OS) and 90.2% (BCSS). *Table 1* shows similarities in clinical characteristics such as mode of referral, tumour size, and prognostic stage between patients who underwent OBCS + RTx and Mx ± RTx, whereas numbers of overall and cancer-specific deaths were more similar between patients who underwent sBCS + RTx and OBCS + RTx. Superior OS and BCSS were observed after OBCS + RTx compared with Mx ± RTx via Kaplan–Meier survival analysis using the log rank test (*Fig. 3*). Superior OS and BCSS remained significant after adjusting for patient demographics, socio-economic deprivation, tumour characteristics, and treatment adjuncts (*Table 5*).

**Table 5:** Multivariable analysis using Cox regression to compare oncoplastic breast-conserving surgery + radiotherapy and mastectomy ± radiotherapy—HRs of overall survival and breast cancer-specific survival by treatment modality adjusted stepwise for patient demographics, socio-economic deprivation, tumour characteristics, staging, and treatment adjuncts

	Deaths	HR (95% c.i.), *P*			
		Model A	Model B	Model C	Model D
**Overall survival**					
OBCS + RTx	37 (10.3)	1 (reference)	1 (reference)	1 (reference)	1 (reference)
Mx + RTx	926 (31.4)	2.38 (1.71,3.31), <0.001	2.26 (1.63,3.15), <0.001	1.70 (1.14,2.51), 0.008	1.72 (1.16,2.55), 0.007
Mx − RTx	734 (31.5)	2.01 (1.44,2.80), <0.001	1.89 (1.35,2.64), <0.001	2.48 (1.67,3.67), <0.001	2.21 (1.49,3.27), <0.001
**Breast cancer-specific survival**					
OBCS + RTx	26 (7.2)	1 (reference)	1 (reference)	1 (reference)	1 (reference)
Mx + RTx	604 (20.5)	2.51 (1.69,3.72), <0.001	2.42 (1.63,3.60), <0.001	1.72 (1.05,2.83), 0.031	1.74 (1.06,2.86), 0.028
Mx − RTx	246 (10.5)	1.28 (0.85,1.92), 0.243	1.22 (0.81,1.84), 0.341	2.06 (1.24,3.42), 0.005	1.89 (1.13,3.14), 0.015

Values are *n* (%) unless otherwise indicated. Model A: adjusted for age, year of diagnosis, and mode of referral. Model B: adjusted for the same variables as model A + socio-economic deprivation. Model C: adjusted for the same variables as model B + tumour grade, tumour size, receptor profile, and lymph node status (pathological staging for primary surgery patients and clinical staging for neoadjuvant chemotherapy patients). Model D: adjusted for the same variables as model C + treatment adjuncts (chemotherapy, biological therapy, and/or endocrine therapy). OBCS, oncoplastic breast-conserving surgery; RTx, radiotherapy; MTx, mastectomy.

**Fig. 4:**
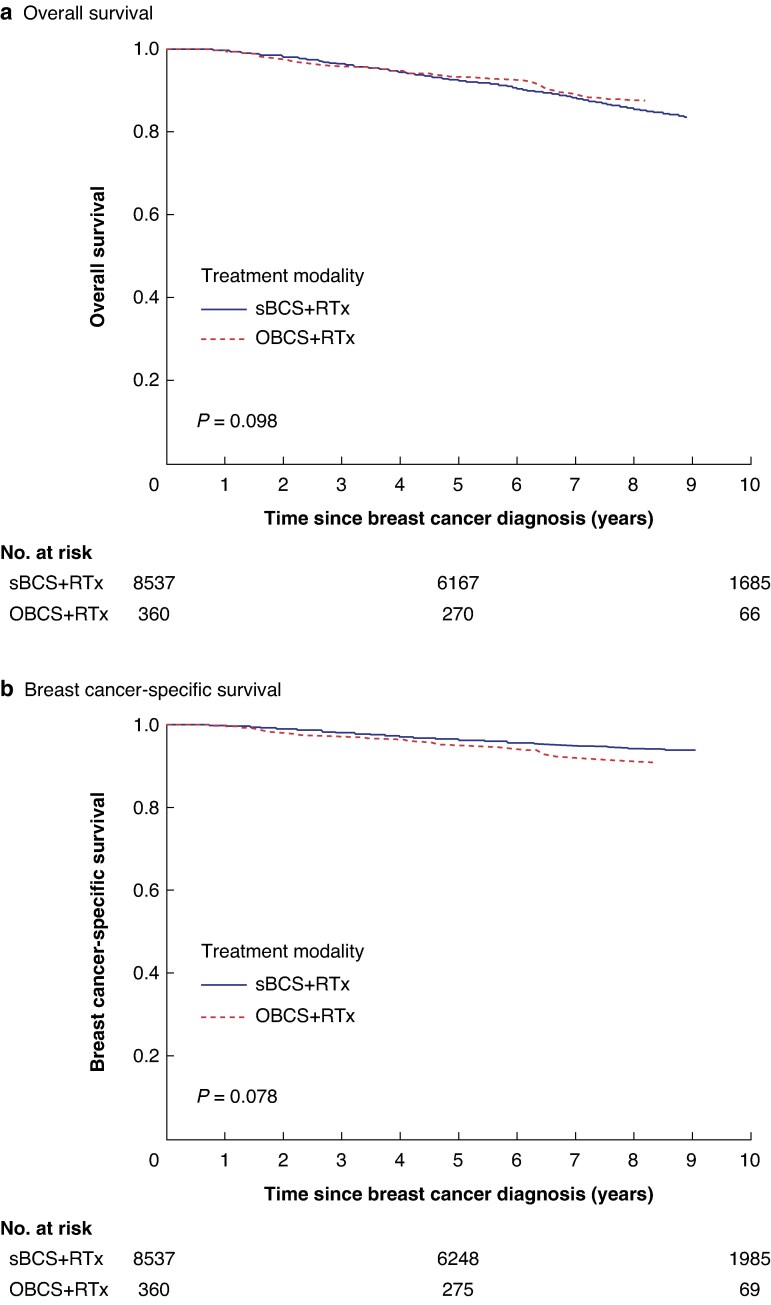
Kaplan–Meier survival curves comparing standard breast-conserving therapy + radiotherapy and oncoplastic breast-conserving surgery + radiotherapy **a** Overall survival. **b** Breast cancer-specific survival. sBCS, standard breast-conserving surgery; RTx, radiotherapy; OBCS, oncoplastic breast-conserving surgery.

Additional analysis comparing sBCS + RTx and OBCS + RTx (8897 patients) demonstrated no significant difference in OS or BCSS, both in the crude analysis (*Fig. 4*) and after adjustment for patient demographics, socio-economic deprivation, tumour characteristics, staging, and treatment adjuncts (OS: HR 0.78 (95% c.i. 0.53 to 1.15), *P* = 0.207; BCSS: HR 0.94 (95% c.i. 0.57 to 1.55), *P* = 0.815). The findings were strengthened by further subgroup analysis comparing survival outcomes after sBCS + RTx and Mx ± RTx (13 822 patients) (*Table S1* and *Fig. S1*).

## Discussion

This large prospective population cohort study demonstrates superior OS and BCSS associated with BCS + RTx compared with Mx ± RTx after adjustment for patient demographics, socio-economic deprivation, mode of referral, tumour characteristics, staging, and treatment adjuncts in the West of Scotland population. In addition, it demonstrates similar survival benefit amongst a subgroup of patients undergoing OBCS + RTx compared with Mx ± RTx. Poor recruitment into the MIAMI trial demonstrated the non-feasibility of conducting an RCT comparing BCS and Mx^23^. Therefore, evidence relies on large population-based registry studies of unselected patients. Comparison of survival outcomes across different regions, where treatment standards may vary, add to overall knowledge and understanding. This is the first UK-based population cohort study comparing survival after breast conservation and Mx. The findings support previous evidence that BCS + RTx compared with Mx ± RTx is associated with superior survival, after adjustment for other known prognostic factors^7–13^.

As OBCS is now the standard of care across the UK, it is imperative to understand how this procedure may impact survival outcomes for patients with breast cancer. A nationwide Scottish audit previously demonstrated that OBCS represents a niche between standard breast conservation and Mx^24^. Therefore, the present study was inclusive of oncoplastic breast conservation procedures, which has not been the case in previously published literature on the survival advantage of breast conservation over Mx. It has been previously shown that patients treated with OBCS in Glasgow breast units have significantly better OS and BCSS compared with those treated with Mx when tumour and treatment characteristics are the same (OS: 98.1% *versus* 84.6%, HR 5.31 (95% c.i. 1.65 to 17.03), *P* < 0.001; BCSS: 99% *versus* 89.4%, HR 5.5 (95% c.i. 1.32 to 22.91), *P* < 0.001)^25^. Similarly, in the present study, superior OS and BCSS were observed after OBCS compared with Mx ± RTx and the benefit was maintained after adjusted analysis. These results suggest that patients who are treated with breast conservation using oncoplastic techniques have a better prognosis compared with those who undergo Mx; hence converting Mx into breast conservation—when feasible—with oncoplastic techniques improves prognoses.

The literature varies with regard to the association of patient age and BCSS after BCS. Three of the largest studies demonstrate superior BCSS after BCS in those over 50 years old^26–28^, whereas others report superior survival across all age categories^29,30^. The present study only found superior BCSS associated with conservation surgery in those aged greater than or equal to 70 years at diagnosis. This is an interesting observation as it is known that older women are more likely to undergo Mx, unexplained by clinicopathological parameters^31^, raising the question of patient and surgical decision-making. In the present study, an even distribution of treatment modalities in those aged greater than or equal to 70 years at diagnosis was observed, with 48% of patients in the cohort undergoing BCS/OBCS and 52% of patients in the cohort undergoing Mx. However, within the BCS group, those aged greater than or equal to 70 years at diagnosis were more likely to undergo sBCS than OBCS (17.6% *versus* 6.1%).

Breast cancers detected through screening are associated with a better prognosis, with increased detection of early, low-grade, and smaller hormone-positive tumours^12,27^. Despite this, only two studies account for mode of referral in their analysis^12,30^. Superior OS (HR 0.66 (95% c.i. 0.59 to 0.75), *P* < 0.001) and BCSS (HR 0.63 (95% c.i. 0.52 to 0.77), *P* < 0.001) were identified in patients presenting through the screening pathway compared with symptomatic patients, consistent with these two previous studies. Superior survival outcomes associated with conservation surgery were maintained after adjustment for mode of referral.

The present study identified inferior OS (HR 1.46 (95% c.i. 1.29 to 1.65), *P* < 0.001) and BCSS (HR 1.34 (95% c.i. 1.11 to 1.60), *P* = 0.002) for those patients living in the most deprived areas, in line with a large, population study from Sweden^11^, albeit using different markers for socio-economic deprivation. Other studies have not adjusted for socio-economic status. Patients living in more deprived areas are less likely to engage in screening programmes, and present later with more advanced disease, which may mean Mx is the only suitable surgical treatment. In addition, these patients are less likely to comply with endocrine or biological therapy, which may also impact survival outcomes. It must also be acknowledged that a higher proportion of patients from the most affluent areas underwent OBCS (25.1%), with a lower proportion from the most deprived areas (19.3%), suggesting a degree of selection bias and a limitation of the present study.

Many previous studies have only included patients with T1–T2 disease to enable fair comparison between conservation surgery and Mx^10,11,13^. Evidence for survival outcomes for T3 tumours is less well described^8^ and, to the authors’ knowledge, there are no studies including T4 tumours. Although larger tumour size may be an indication for Mx, everyone now works in an era in which neoadjuvant treatments can be given to reduce tumour size and/or oncoplastic techniques can be used to enable conservation surgery^32^. The present study included all tumour sizes (T1–T4) and survival outcomes were maintained in adjusted analysis. Similarly, node-positive disease was associated with inferior OS and BCSS, but superior BCSS after conservation surgery remained significant in multivariable analysis, supporting previous evidence^11,13,30^. In terms of receptor profile expression, inferior BCSS was found to be associated with ER+HER2+ (HR 1.51 (95% c.i. 1.12 to 2.02), *P* = 0.007) and ER−HER2− (HR 1.41 (95% c.i. 1.01 to 1.97), *P* = 0.042) tumours; however, this did not impact survival outcomes in adjusted analysis.

A larger surgical insult is associated with an increased risk of postoperative complications and has been associated with inferior survival in other cancer subtypes^33,34^. de Boniface *et al*.^35^ analysed the impact of postoperative complications on survival in breast cancer. Major postoperative complications were more common after Mx with or without reconstruction (7.3% and 4.3% respectively) compared with BCS (2.3%), with associated inferior OS and BCSS after adjustment for co-morbidity and clinicopathological confounders. In the Scottish audit of oncoplastic breast conservations a trend towards decreased disease-free survival at 5 years was found in patients with major postoperative complications after OBCS; however, this was not statistically significant (86.11% *versus* 92.1%, *P* = 0.236)^36^.

Surgical trauma activates a systemic stress response with immunosuppressive effects and pro-inflammatory pathways, which may in turn promote growth of any residual local tumour cells, circulating tumour cells, and micrometastases^37^. Postoperative complications are thought to add to, prolong, and accelerate these processes and, in addition, can cause delays to adjuvant systemic treatments^37^. The underlying mechanism is multifactorial and not yet fully understood^38,39^. Although information on postoperative complications is not available for the patients in the present study, postoperative complications may have contributed to the inferior survival outcomes seen in the Mx ± RT cohort and may also explain why Mx + RTx was associated with the lowest BCSS in unadjusted analysis.

Despite improved overall and relative survival after BCS in all patient subgroups, van Maaren *et al*.^10^ only found significantly improved 10-year disease-free survival in the T1 N0 subgroup. Studies assessing the relationship between treatment modality and disease recurrence are lacking, particularly for more advanced disease, and further research is required. Disease recurrence causes significant patient anxiety—better understanding of risk factors is an important research priority for both patients and clinicians^40^.

This large, population-based study demonstrates survival benefit after conservation surgery coupled with RTx compared with Mx ± RTx. All data were collected prospectively and the selected study interval allows for up to 13.6 years of follow-up. Tumours of all sizes were included and it was possible to adjust for any potential survival benefit associated with tumours detected through screening. In addition, similar survival benefit amongst patients undergoing OBCS + RTx compared with Mx ± RTx is shown, which has not been demonstrated in the existing literature.

The study data set did not include information about the reasons behind choices made around treatment modalities for each of the individual clinical situations. Also, information regarding patient co-morbidities was not available; however, it was possible to adjust for socio-economic deprivation, which might be seen as a proxy for multi-morbidity. Selection bias associated with OBCS has already been mentioned as a limitation of the present study—patients from more affluent areas are likely to be better educated with improved health literacy and fewer co-morbidities, which can affect survival rates regardless of treatment modality. This may also explain the differences observed for OS comparing sBCS and OBCS (*Fig. 4*), albeit this was not statistically significant. In addition, data on RTx site(s) and dosage were lacking and disease recurrence is not collected as part of the National Cancer Registry. The present study found no impact of chemotherapy on BCSS—one explanation for this may be that the earlier cohort pre-dates routine genomic testing for ER+HER2− tumour subtypes in determining chemotherapy benefit in addition to endocrine therapy^41^. Future studies should: explore the impact of patient co-morbidity on survival outcomes after different treatment modalities; account for different RTx regimens; and consider if different treatment modalities impact disease recurrence in breast cancer.

Both OS and BCSS are superior after BCS + RTx and OBCS + RTx compared with Mx ± RTx, after adjustment for other known prognostic factors, in this Scottish population-based study. The findings should inform discussion of surgical treatment options with patients with breast cancer.

## Supplementary Material

zraf002_Supplementary_Data

## Data Availability

The authors are willing to make their data, analytical methods, and study materials available to other researchers upon reasonable request to the corresponding author, including relevant ethical and legal permissions. The analysis presented was not pre-registered.
