# Supplementary material for: Survival after standard or oncoplastic breast-conserving surgery versus mastectomy for breast cancer
Source: BJS Open. 2025 Mar 19;9(2):zraf002. doi: 10.1093/bjsopen/zraf002 (PMC11920510; doi:10.1093/bjsopen/zraf002)
Supplement: zraf002_Supplementary_Data [file zraf002_supplementary_data.docx]

# Breast conservation surgery – including oncoplastic breast surgery – is associated with increased survival in comparison to mastectomy in a West of Scotland population-based study with long-term follow-up

Mhairi Mactier^1,2^, James Mansell^3^, Laura Arthur^4^, Julie Doughty^3^, Laszlo Romics^3^

1. Wolfson Wohl Cancer Research Centre, College of Medicine and Veterinary Science, University of Glasgow, Glasgow, UK
2. General Surgery Department, Golden Jubilee National Hospital, Clydebank, UK
3. General Surgery Department, Gartnavel General Hospital, Glasgow, UK
4. General Surgery Department, Royal Alexandria Hospital, Paisley, UK

**Corresponding author**

Miss Mhairi Mactier

Wolfson Wohl Cancer Research Centre

College of Medicine and Veterinary Science

University of Glasgow

Garscube Estate

Switchback Road, Bearsden

G61 1BD

[2971527M@student.gla.ac.uk](mailto:2971527M@student.gla.ac.uk)

[mhairi.mactier2@nhs.scot](mailto:mhairi.mactier2@nhs.scot)

ORCID: 0000-0001-6177-135X

Twitter/X: @MactierMhairi

**Supplementary Materials - Index**

| **Supplementary Methods** |  |
| --- | --- |
| *N/A* |  |
| **Supplementary Results** |  |
| *N/A* |  |
| **Supplementary Appendixes** |  |
| *N/A* |  |
| **Supplementary Figures and Tables** |  |
| Table s1: Multi-variant analysis using Cox regression comparing sBCS+RTx to Mx±RTx - Hazard ratios of overall survival and breast cancer-specific survival by treatment modality adjusted stepwise for patient demographics, socio-economic deprivation, tumour characteristics, staging and treatments adjuncts | *Page 3* |
| Figure s1: Kaplan-Meier survival curve comparing sBCS+RTx and Mx±RTx for overall survival (A) and breast cancer-specific survival (B) | *Page 4* |
| **References**  *N/A* |  |
|  |  |

**Supplementary Methods**

**N/A**

**Supplementary Results**

**N/A**

**Supplementary Appendixes**

**NA**

**Supplementary Figures and Tables**

Table s1: Multi-variant analysis using Cox regression comparing sBCS+RTx to Mx±RTx - Hazard ratios of overall survival and breast cancer-specific survival by treatment modality adjusted stepwise for patient demographics, socio-economic deprivation, tumour characteristics, staging and treatments adjuncts

*Model A: Adjusted for age, year of diagnosis and mode of referral*

*Model B: Adjusted for same variables as model A + socio-economic deprivation*

*Model C: Adjusted for same variables as model B + tumour grade, tumour size, receptor profile and lymph node status (pathological staging for primary surgery patients; clinical staging for NACT patients)*

*Model D: Adjusted for same variables as model C + treatment adjuncts (Y/N chemotherapy, biologics AND/OR endocrine therapy*

|  | **No. Deaths (%)** | **Hazard Ratio (95% CI) p-value** | | | |
| --- | --- | --- | --- | --- | --- |
|  |  | **Model A** | **Model B** | **Model C** | **Model D** |
| **Overall Survival**   - **sBCS+RTx** - **Mx+RTx** - **Mx-RTx** | 1265 (14.8)  926 (31.4)  734 (31.5) | 1 [Reference]  1.94(1.77-2.11) p<0.001  1.58(1.44-1.74) p<0.001 | 1 [Reference]  1.95(1.79-2.13) p<0.001  1.59(1.45-1.75) p<0.001 | 1 [Reference]  1.26(1.12-1.42) p<0.001  1.65(1.48-1.84) p<0.001 | 1 [Reference]  1.29(1.15-1.45) p<0.001  1.56(1.40-1.73) p<0.001 |
| **Breast Cancer Specific Survival**   - **sBCS+RTx** - **Mx+RTx** - **Mx-RTx** | 450 (5.3)  604 (20.5)  246 (10.5) | 1 [Reference]  3.32(2.84-3.66) p<0.001  1.63(1.39-1.91) p<0.001 | 1 [Reference]  3.27(2.88-3.71) p<0.001  1.64(1.40-1.92) p<0.001 | 1 [Reference]  1.53(1.30-1.80) p<0.001  1.79(1.48-2.16) p<0.001 | 1 [Reference]  1.54(1.30-1.81) p<0.001  1.69(1.40-2.04) p<0.001 |

Figure s1: Kaplan-Meier survival curve comparing sBCS+RTx and Mx±RTx for overall survival (A) and breast cancer-specific survival (B)

**
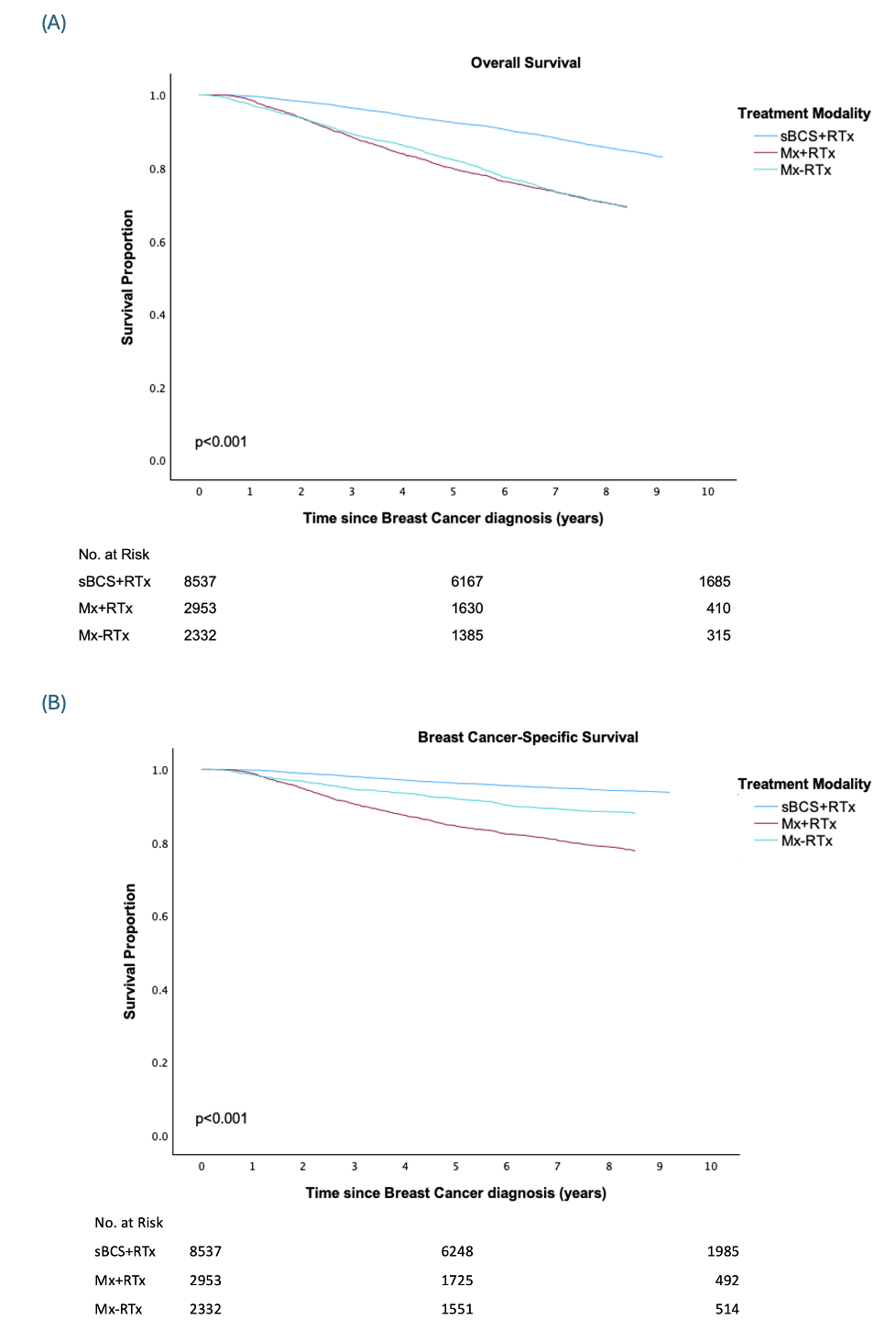
**

**References**

**NA**
